# Perceptions about mental illness among general practitioners

**DOI:** 10.1186/s13033-019-0284-9

**Published:** 2019-04-13

**Authors:** Mª Carmen Castillejos Anguiano, Antonio Bordallo Aragón, David Aguilera Fernández, Berta Moreno Küstner

**Affiliations:** 1 Andalusian Group of Psychosocial Research (GAP), Department of Personality, Assessment and Psychological Treatment, University of Malaga, Campus Teatinos, Malaga, Spain; 2 Clinical Management Unit of Mental Health of the Regional Hospital of Malaga, Andalusian Health Service, Avda del Hospital Civil S/N, Paseo Limonar, Malaga, Spain; 3 Biomedical Research Institute of Malaga (IBIMA), Malaga, Spain

**Keywords:** Primary care, Schizophrenia, Stigma, Collaborative model

## Abstract

**Background:**

General practitioners (GPs) play an important role in the physical care of patients with severe mental illness, so our aim was to analyse the relationships between GPs’ sociodemographic status and worked-related variables and their perceptions about mental illness.

**Methods:**

A descriptive, cross-sectional study was conducted in the Clinical Management Unit of Mental Health (CMU-MH) of the Regional Hospital of Malaga (Spain). The eligible population comprised all GPs working in the 13 primary care centres (PCCs) in the hospital’s catchment area during the study period. GPs were interviewed to collect data on their attitudes to and knowledge of mental illness, psychiatry and the local mental health team, as well as their sociodemographic status, professional qualifications and experience. Bivariate analysis was carried out.

**Results:**

145 GPs answered the questionnaire (77%). ANOVA revealed that most of the PCCs with the best relationship with their mental health team and best attitude to mental illness were in the Central Community Mental Health Unit, which operated a collaborative model of care.

**Conclusions:**

These results indicated that GPs who worked more closely with their specialist mental health team had a better perception of their relationship with the mental health centre and less stigmatisation in regard to mental illness.

## Background

It is widely known that patients with mental illness experience discrimination and stigmatisation [1–3]. There has been a lot of research into self-stigmatisation by individuals with mental illness [4, 5], which is often due to societal stigmatisation of mental illness [6, 7].

One would expect health professionals to have a more positive attitude to mental illness and the mentally ill because of their professional knowledge, but several studies have shown that this is not the case [8, 9]. A recent systematic review concluded that older general practitioners (GPs) had a more negative attitude to patients with schizophrenia [10]. It has been shown that GPs’ stigmatisation of patients with mental illness depends on their level of experience of such patients, so that the more experience they have, the less stigmatisation they exhibit [11]. Comparisons of the attitudes of different categories of health professional have shown that GPs stigmatise mental illness more than psychiatrists do [12–14].

Health professionals’ perception of mental illness and their attitude to the mentally ill could influence their decisions in daily practice [15]. Moreover, health professionals’ negative attitude is detected by patients [16] and may be a factor in the low rate of engagement of patients with serious mental illness with healthcare services [14].

A recent study carried out in the United States showed that GPs faced significant barriers to providing good care to patients with mental illness: lack of time and resources and lack of confidence [11, 17].

GPs play an important role in the care of patients with severe mental illness (SMI) because these patients have higher rates of mortality and morbidity from physical health problems than the general population [18–20] and because GPs are the main point of entry into the Spanish health system. We therefore aimed to analyse the relationships between GPs’ sociodemographic status, work-related variables and their perceptions regarding mental illness. These perceptions will be defined as the level of satisfaction of GPs with their relationship with the community mental health centre, their erroneous beliefs, stigmatisation and attitudes regarding mental illness and their perception of their level of training in mental health, schizophrenia and other psychotic disorders.”.

## Material and methods

This was a descriptive, cross-sectional study.

### Study area and temporal scope

The study was carried out in the CMU-MH of the Regional Hospital of Malaga, with two Community Mental Health Units (CMHUs), Central and Northern, which together covered a population of 315,159. The Central CMHU included 6 PCCs: Alameda-Perchel, Victoria, Limonar, El Palo, Colmenar and el Rincón de la Victoria; and the Northern CMHU included 7 PCCs: Trinidad, Nueva Malaga, Miraflores, Palma-Pamilla, Ciudad-Jardín, Capuchinos and Carlinda.

The study information was collected from January 1, 2008 to July 1, 2011.

### Eligible population and sample

The eligible population comprised 188 GPs working in the 13 PCCs in the catchment area of CMU-MH of the Malaga Regional Hospital during the study period.

### Questionnaire and study variables

We used the Primary Care Physicians and Mental Health Questionnaire (MAPSAM-14), which had been validated by the research team [21], to assess perceptions of GPs towards mental health.

So our dependent variables were scores on the three MAPSAM-14 scales, (1) Relationship: level of satisfaction of GPs with their relationship with the community mental health centre (range 7–15; higher scores indicated greater satisfaction with the relationship); (2) Beliefs: this touched upon erroneous beliefs, stigmas and attitudes regarding mental illness (range 5–12; higher scores indicate more erroneous beliefs and greater stigmatisation); (3) Training: the GPs’ perception of their level of training in mental health, schizophrenia and other psychotic disorders (range 5–15; higher scores indicate greater perceived adequacy of training).

The independent variables were age; gender; years in the current place of work ( ≤ 3 years; > 3 years); specialisation training as a GP (yes/no); accredited training as a tutors (yes/no); to have any residential training students (yes/no); size of patient list ( ≤ 1500; > 1500), PCC affiliation (Alameda-Perchel, Victoria, Limonar, El Palo, Colmenar, el Rincón de la Victoria, Trinidad, Nueva Malaga, Miraflores, Palma-Pamilla, Ciudad-Jardín, Capuchinos and Carlinda.). These variables were obtained using a self-report questionnaire.

### Data analysis

Bivariate analysis of the relationships between the sociodemographic and work-related variables and the MAPSAM-14 variables (Relationship, Beliefs and Training) was carried out. For the independent dichotomous qualitative variables a Student's t-test was used. And for the independent qualitative polytomous variables, analysis of variance (ANOVA) was used. Differences were considered significant at *p* < 0.05.

The statistical program SPSS statistics 22 was used for the construction of the database, the descriptive analyses and for the bivariate analysis.

## Results

There were 188 GPs serving the catchment area of Malaga regional hospital, of whom 145 answered the questionnaire, a response rate of 77%.

The mean age of the sample was 50.5 (95% CI 49.5–51.5; range 35–63) and the median age was 51.5 years (range 35–6 and 64.7% was male. Mean professional experience was 7.2 years (95% CI 5.3–9.1; range 0–30). Forty-four percent of participating GPs had hospital resident training, 64.7% were not accredited training tutors and 60.8% had not led a hospital doctor team in the last 3 years. The mean patient list size was 1608 (95% CI 1538.2–1678.8; median = 1500; range 900–2500).

Turning to the MAPSAM-14 variables, the mean scores were as follows: the level of satisfaction of GPs with their relationship with the community mental health centre [Relationship] was M = 12.51 (95% CI 12.16‒12.87), with the highest level of satisfaction being M = 15; the dimension [Beliefs] that touched upon erroneous beliefs, stigmas and attitudes regarding mental illness was M = 8.04 (95% CI 7.79–8.28), with the highest level of stigmatisation being M = 12; and the dimension [Training] that measured the perception of GPs of their level of training in mental health, schizophrenia and other psychotic disorders was M = 8.61 (95% CI 6.43–10.80), with the highest rate being M = 15, which indicates greater perceived adequacy of training.

As result of the bivariate analysis, the only dichotomous variable related to the level of satisfaction of GPs with their relationship with the community mental health centre was size of patient list: having a list of more than 1500 patients was associated with a better level of satisfaction with their relationship with the community mental health centre (*p* = 0.034) (Table 1).

**Table 1:** Student's *t*-tests for GPs’ sociodemographic status, professional experience and qualifications

	t	df	p value	Mean differences	Standard error differences	95% confidence interval	
						Lower limit	Upper limit
D1: Relationship							
Gender	− 1.739	140	0.084	− 0.626	0.360	− 1.338	0.086
Years in the current place of work	− 0.074	91	0.941	− 0.033	0.443	− 0.912	0.846
Specialisation training as a GP	0.882	137	0.379	0.324	0.368	− 0.403	1.501
Accredited training as a tutors	0.515	140	0.607	0.194	0.377	− 0.552	0.941
To have any residential training students	0.589	140	0.557	0.221	0.374	− 0.519	0.960
Size of patient list	− 2.139	140	0.034	− 0.780	0.365	− 1.500	− 0.059
D2: Beliefs							
Gender	0.882	140	0.379	0.224	0.254	− 0.278	726
Years in the current place of work	1.558	91	0.123	0.504	0.323	− 0.139	1.146
Specialisation training as a GP	− 1.462	137	0.146	− 0.372	0.255	− 0.876	0.131
Accredited training as a tutors	− 1.264	140	0.208	− 0.332	0.263	− 0.852	0.187
To have any residential training students	− 1.736	140	0.085	− 0.450	0.259	− 0.963	0.063
Size of patient list	1.391	140	0.166	0.358	0.257	− 0.151	0.867
D3: Training							
Gender	0.585	143	0.559	1.307	2.232	− 3.105	5.718
Years in the current place of work	− 1.419	93	0.159	− 3.896	2.746	− 9.348	1.557
Specialisation training as a GP	− 0.413	140	0.680	− 0.936	2.265	− 5.414	3.543
Accredited training as a tutors	− 1.285	143	0.201	− 2.982	2.321	− 7.570	1.606
To have any residential training students	− 1.109	142	0.269	− 2.115	1.907	− 5.885	1.654
Size of patient list	1.635	143	0.107	4.466	2.731	− 1.002	9.933

The ANOVA revealed differences between PCCs GPs affiliations with respect to the level of satisfaction with their relationship with the community mental health centre and beliefs about mental illness (both *p*s < 0.001). The PCCs where GPs’ perception of the relationship with the local mental health team was best were El Rincón de la Victoria and El Palo. The PCCs where GPs had less stigmatisation regarding mental illness were Victoria, El Palo, El Rincón de la Victoria, Limonar, Alameda-Perchel and Nueva Malaga (Table 2); all these PCCs were linked with the Central CMHU except for Nueva Malaga.

**Table 2:** ANOVA of primary care centre differences in relationship with the local mental health team, beliefs about mental illness and perceptions of the adequacy of training in mental health

	Sum of squares	df	Root mean square	F	p value
D1: Relationship					
Between groups	186.835	12	15.570	4.360	< 0.001
Within groups	460.637	129	3.571		
Total	647.472	141			
D2: Beliefs					
Between groups	90.570	12	7.548	4.303	< 0.001
Within groups	226.254	129	1.754		
Total	316.824	141			
D3: Training					
Between groups	1957.781	12	163.148	0.914	0.535
Within groups	23,568.591	132	178.550		
Total	25,526.372	144			

## Discussion

This study analysed the relationships between the three MAPSAM-14 dimensions (level of satisfaction of GPs with their relationship with the community mental health centre, their erroneous beliefs, stigmatisation and attitudes regarding mental illness and their perception of their level of training in mental health, schizophrenia and other psychotic disorder) and GPs’ age, gender and professional experience and qualifications.

The only variable associated with GPs’ relationship with their local mental health teams was the size of their patient list; those GPs with a longer list ( > 1500) perceived that they had a better relationship with the mental health team, perhaps because they had more contact with it and, therefore, this fact could be valued by the GPs as a more effective communication and relationship.

None of the sociodemographic or work-related variables was associated with GPs’ beliefs about mental illness, which conflicts with the results of Rojas-Vistorte et al. and Lam et al. [10, 22]. They found that GPs who stigmatised mental illness tended to be more experienced, female, work at hospital level and not to have relatives or friends affected by mental disorders.

Finally, GPs perception of the adequacy of their training in mental health problems was not associated with any of the sociodemographic or work-related variables.

Our most important finding, however, was that GPs’ perception of their relationship with the local mental health team and their beliefs about mental illness varied according to their PCC affiliation. The GPs which reported the best relationship with the community mental health centre were all working in PCCs located in the catchment area of the Central CMHU. Similarly, GPs affiliated to the six teams with the lowest Belief scores (i.e. less erroneous beliefs, stigmas and attitudes regarding mental illness) all but one (Nueva Malaga) belonged to the Central CMHU catchment area. At the time of the study the Central CMHU had been working collaboratively with GPs for over 15 years, whereas the Northern CMHU had more traditional working relationships with GPs. This suggests that collaborative working improves communication and relationships between specialist mental health teams and GPs. It may also help to minimise misunderstanding of mental illness amongst GPs, because greater contact between GPs and mental health teams may increase GPs’ knowledge and understanding of mental illness; this relationship between contact with mental health services and knowledge of mental illness has in fact been observed in the general population [23]. Probably these aspects favour better care for patients with mental illness from Primary Care. At present, new, more collaborative relationship between primary and secondary care are being introduced in order to provide patients with mental illness with better, more holistic care [24]. Collaborative ways of working have been shown to improve the care of patients with severe mental illness [25–27]. A study carried out in a rural area showed that patients preferred services where there was an emphasis on collaboration between primary care and specialist mental health services [28].

### Strengths and limitations

- This study analysed multiple associations between GPs’ characteristics and their attitude to and knowledge of mental illness and their relationship with their local mental health team.
- The study was carried out in a clinical mental health management unit with considerable experience of working collaboratively with primary care practitioners; the unit concerned is one of the pioneers in this area in Andalucia.
- The study was carried out in a wide catchment area, including participants from 13 primary care centres. However, we could not consider differences between rural and urban PCCs as we had only one small PCC in a rural area.
- For unknown reasons not all GPs in the study area participated, and in some cases, the number of GPs per centre was very low. However, in general, the participation rate was high.
- We should be aware that there could be a bias concerning GPs who answer the questionnaire as they could have a better perception of their level of training in mental health or a more positive attitude towards mental illness.
- We did not analyse nursing staff, although they are very involved in the treatment of patients with mental illness.

## Conclusion

In this study we analysed relationships between multiple GP characteristics and GPs’ attitudes to mental illness, but our main finding was that GPs working at PCCs in the catchment area of Central CMHU, which had much greater experience of working collaboratively with GPs than the other CMHU in the study, perceived themselves to have a better relationship with their local mental health centre and less stigmatisation regarding mental illness.

## Relevance for clinical practice

Due to the results obtained, and given the important role that GPs play in caring for the physical health of patients with severe mental illness, we strongly recommend that more collaborative relationships between primary care teams and specialist mental health teams are widely implemented, in order to improve overall healthcare for people with mental illness.
